# The Impact of COVID-19 on Travel Mode Choice Behavior in Terms of Shared Mobility: A Case Study in Beijing, China

**DOI:** 10.3390/ijerph19127130

**Published:** 2022-06-10

**Authors:** Xiaoyu Zhang, Chunfu Shao, Bobin Wang, Shichen Huang

**Affiliations:** 1 Key Laboratory of Transport Industry of Big Data Application Technologies for Comprehensive Transport, Beijing Jiaotong University, Beijing 100044, China; 18114055@bjtu.edu.cn (X.Z.); 15114225@bjtu.edu.cn (S.H.); 2 Department of Mechanical Engineering, Université Laval, Quebec, QC G1V 0A6, Canada; bobin.wang@gmc.ulaval.ca

**Keywords:** shared mobility, COVID-19, travel mode choice, stated preference experiment, latent class analysis, nested logit model

## Abstract

Shared mobility is growing rapidly and changing the mobility landscape. The COVID-19 pandemic has complicated travel mode choice behavior in terms of shared mobility, but the evidence on this impact is limited. To fill this gap, this paper first designs a stated preference survey to collect mode choice data before and during the pandemic. Different shared mobility services are considered, including ride hailing, ride sharing, car sharing, and bike sharing. Then, latent class analysis is used to divide the population in terms of their attitudes toward shared mobility. Nested logit models are applied to compare travel mode choice behavior during the two periods. The results suggest that shared mobility has the potential to avoid the high transmission risk of public transport and alleviate the intensity of private car use in the COVID-19 context, but this is limited by anxiety about shared spaces. As the perceived severity of the pandemic increases, preference for ride hailing and ride sharing decreases, and a price discount for ride hailing is more effective than that for ride sharing at maintaining the ridership despite the impact of COVID-19. These findings contribute to understanding the change in travel demand and developing appropriate strategies for shared mobility services to adapt to the pandemic.

## 1. Introduction

With the rapid expansion in information and communication technology (ICT), shared mobility, i.e., on-demand ride services, has become increasingly prevalent in metropolitan regions across 66 countries [1]. As an example of the creative economy in the mobility landscape, shared mobility presents advantages including a short waiting time, increased comfort, and provision of door-to-door services [2]. Shared mobility not only shifts the fixed cost of automobile ownership into flexible payments varying with travel time and distance, but also removes difficulties faced involving parking [3]. Different mobility services, such as ride hailing, ride sharing, car sharing, and bike sharing, are being offered by transportation network companies [4]. These emerging services and attitudes toward them influence travel mode choice behavior.

Ride hailing enables users to request a ride and query vehicle trajectories using platforms such as Uber, Lyft, and DiDi. Ride hailing comprises three types of vehicle reservation services: ride hailing basis, ride hailing express, and ride hailing premier [2]. As the basic type of ride hailing, ride hailing basis effectively matches riders and available taxis through centralized dispatching, and thus alleviates deadhead driving seeking passengers. Ride hailing express is more economical and the drivers are private car owners. Ride hailing premier provides quality service with expensive vehicles and well-trained chauffeurs.

Ride sharing emphasizes making full use of in-vehicle space, including through ride splitting and car pooling [5,6]. With ride splitting, travelers on similar routes are matched to the same vehicle by Uber, DiDi, and AmigoExpress, and spend less money than when ride hailing. With car pooling, drivers drop people off on the way to a destination and passengers share the cost of the journey with the driver.

Car sharing involves a self-service car rental with pricing units in hours or minutes and is offered by companies such as Zipcar, Car2Go, and GoFun [7]. In this study, car sharing particularly refers to station-based cars. Many parking stations are located in car sharing regions, and users can pick up and return vehicles to different nearby stations as required. The only non-motorized form of shared mobility is bike sharing, consisting of almost 18 million bicycles in more than 2100 cities worldwide as of early 2020 [8]. 

Since late 2019, the COVID-19 pandemic has become an international public health emergency. On 23 February 2021, the global cumulative number of confirmed cases reached 110.7 million, with the death toll surpassing 2.4 million [9]. The perceived severity of the pandemic has complicated travel mode choice behavior in terms of shared mobility. The ridership of shared mobility was limited by home quarantines and anxiety regarding entering shared spaces [10]. However, new mobility services may play a vital role in post-pandemic travel industry recovery with advantages such as reliability, sustainability, and less in-vehicle crowding [11]. Additionally, COVID-19 may impact travel mode choice behavior regarding different mobility services based on their respective characteristics.

Existing studies have discussed different factors that influence shared mobility use [12,13], but the evidence is limited on the impact of COVID-19. Additionally, most studies mainly pay attention to specific types of shared mobility services [3]. Since different mobility services have different advantages, a comprehensive analysis of their use is beneficial in excavating more personalized and detailed demand.

This study aims to compare travel mode choice behavior in terms of shared mobility before and during the COVID-19 pandemic and determine the price discount needed to maintain the ridership of specific mobility services despite the impact of COVID-19. Different shared mobility services are considered, including ride hailing, ride sharing, car sharing, and bike sharing. A stated preference survey is designed to collect travel mode choice data. Then, latent class analysis is used to divide travelers based on attitudes toward shared mobility use. Nested logit models are applied to explore travel mode choice behavior during the two periods and predict mode split in response to the perceived severity of the pandemic and the effect of a price discount.

Two contributions are made in this study. First, this study provides a better understanding of travel demand changes as a result of the pandemic by comparing travel mode choice behavior during the two periods, which will facilitate effective policy making to optimize post-pandemic travel industry recovery. Second, the study findings contribute to developing appropriate strategies for shared mobility services to adapt to the pandemic and further optimize post-pandemic travel industry recovery.

The remainder of this article is organized as follows. Section 2 provides a brief review of the related literature. Section 3 describes the research framework and modeling approach. Section 4 presents analysis results. Section 5 discusses the key findings. Finally, Section 6 concludes and suggests a future research direction.

## 2. Literature Review

### 2.1. Different Factors That Influence Shared Mobility Use

Previous studies examined various factors that influence shared mobility use, divided into external and internal types. External factors are exogenous to shared mobility ridership, such as socio-demographics (e.g., gender, age, income, education, and car ownership) [12], attitudes [14], and weather [15]. Internal factors include trip-related variables (e.g., trip purpose, trip distance, and time of day) [7] and mode-specific attributes (e.g., travel time, time spent searching for parking, and waiting time) [16]. Table 1 summarizes factors related to different mobility services in the literature.

Ride hailing is mostly used by the post-millennial (ages 18–24) and millennial (ages 25–39) generations [17]. This service is favored by people with higher incomes who are less concerned about convenience, safety, and reliability [21,25]. When there are unplanned subway disruptions, neighborhoods requiring improvement have less availability for ride hailing than advantaged neighborhoods [34]. Moreover, individuals who prioritize a shorter waiting time prefer ride hailing [35]. 

The main motivations for ride sharing are avoiding travel costs and parking issues, preferred by those with lower education, lower income, and non-vehicle owners [23]. The perceived risks of ride sharing are considered less important than the perceived benefits, which include lower cost, convenience, pleasure, exploration, and social identity [26]. Further, the need for independence outweighs safety concerns for some weak populations [36]. Ride splitting is mainly used for shopping and going to restaurants, while car pooling is mainly used for going to airports or railway stations [5].

Car sharing is preferred by environmentalists, used for leisure trips more than commuting [7,30]. Distance to nearest rental site is a limiting factor in car sharing, but it is not as significant when taking into account the inconvenience of public transport [19]. Further, avoiding time spent searching for parking space is valued at 20% higher than on-road travel time [31].

Bike sharing is mostly used for trips and commuting within 30 min [8]. Bike sharing is sometimes a faster alternative for trips within 3 km in prosperous urban areas [37]. The ridership depends on coverage area, parking spots, ease of use, and wearing helmets [38]. Transfer times and travel time when using public transport also influence bike sharing [39]. Moreover, avoiding congestion has a stronger effect than economic and environmental benefits [29].

### 2.2. Travel Mode Choice Analysis during the COVID-19 Pandemic

Some studies have correlated travel mode choice with the COVID-19 pandemic. The ridership in 95% of transit stations has dropped 72.4% on average, and there is a lower decline in ridership in regions with more confirmed cases and deaths [40]. Passengers paying more attention to prevention information perceive taking public transport as less safe [41]. Some travelers still choose public transport for a 15-min trip, but switch to commuting by car as travel time increases [42]. Trapped in heightened anxiety during travel in overcrowded public transport, some commuters intend to purchase a car within a year [43]. Even though overall travel demand decreased, the number of private car users remains high and has increased by 13% during the COVID-19 pandemic [44]. 

The COVID-19 pandemic has brought both opportunities (e.g., a spike in using green mobility) and challenges (e.g., lack of trust among travelers and less money spent on transport) for shared mobility [45]. Public expressions and remarks regarding ride hailing on Twitter have been generally been positive during the COVID-19 pandemic [46]. A large number of carless populations previously used ride hailing for recreational activities. However, such services have been hit hard by anxiety over confined spaces and unfamiliar drivers spreading the coronavirus [47]. To reduce interactions with strangers, car sharing can be an option for those who cannot work remotely and do not have other alternatives to public transport [48]. Moreover, with the shock of the pandemic, people are changing their habits in favor of a healthier and more sustainable lifestyle. The number of people with no experience of bike sharing declined by 49.77%, and regions with more white and Asian residents, and fewer black residents, have been less dependent on bike sharing during the pandemic [49,50].

## 3. Methodology

### 3.1. Research Framework

**Figure 1:**
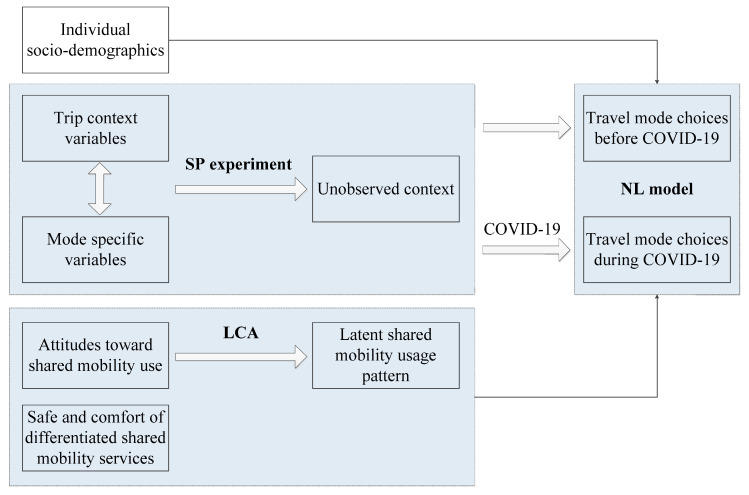
Research framework.

We compared travel mode choice behavior during the two periods to determine the impact of COVID-19 on travel mode choice behavior in terms of shared mobility. Nested logit (NL) models are employed to formulate travel mode choice before and during the pandemic. The stated preference (SP) experiment is designed to enrich observations. Through the latent class analysis (LCA) of attitudes toward shared mobility services, a shared mobility use pattern is constructed and considered as a factor in NL models. The scheme of the research framework is depicted in Figure 1.

### 3.2. Survey and Data

Beijing, the capital of China, is a typical city with intense competition among different shared mobility services, and is thus chosen as our study site. The questionnaire consists of three parts: (1) socio-demographics: gender, age, whether they have school-age children, car ownership, and residential district—respondents are also asked to indicate how they perceive the severity of the pandemic with a scale from 0 to 100; (2) a stated preference (SP) experiment (described in Section 3.2.1); and (3) attitudes toward shared mobility services (more detailed in Section 3.2.3).

#### 3.2.1. Stated Preference Experiment Design

We design a SP experiment to systematically discuss the effects of factors on travel mode choice. Respondents choose from ten travel modes, including three conventional services (public transport, private car, and taxi) and seven shared mobility services (ride hailing basis, ride hailing express, ride hailing premier, ride splitting, car pooling, car sharing, and bike sharing). Here, public transport refers to buses or metros. 

Trip context and mode-specific variables are considered together in the SP experiment. The former includes four trip purposes (commute, leisure, transportation terminal access, and daily routine), four times of the day (morning peak, evening peak, off-peak, and night), and five travel distances (3, 10, 15, 20, and 30 km), covering a full range of travel—short, medium, and long distance.

The mode-specific variables and their ranges are in accordance with real traffic performance, as follows:

(1) Waiting time: according to [51,52], the average bus departure times in Beijing are in the range of 9–21 min, and the longest waiting time in suburban areas is greater than 40 min. Therefore, the waiting time for public transport in our experiment is divided into 8, 15, 25, and 40 min. Additionally, the average waiting time for taxis and ride hailing is 11.9 and 5.6 min [53]. Taking traffic congestion into account, the waiting time for taxis is divided into 10 and 30 min; and the waiting time for ride hailing basis, ride hailing express, ride hailing premier, ride splitting, and car pooling is divided into 5 and 25 min.

(2) Transfer times: considering the road network and metro line distribution, the levels of transfer times for public transport are 0, 1, 2, and 3.

(3) Parking cost: following the charging standard set by [54], the parking costs of private cars are 5 CNY, 12 CNY, and 20 CNY per hour (equal to 0.8, 1.9, and 3 USD).

(4) Detour time: Li et al. 2019 confirmed that ride splitting has an average delay of 10 min, and more than 95% of the delay is within 30 min. Thus the detour time for ride splitting is 10 and 30 min.

(5) Access distance: Jin et al. [7] determined the access distance of car sharing as 0.5–2 km. Based on site coverage in Beijing, this variable can be divided into 0.7, 1.2, and 2 km. 

**Figure 2:**
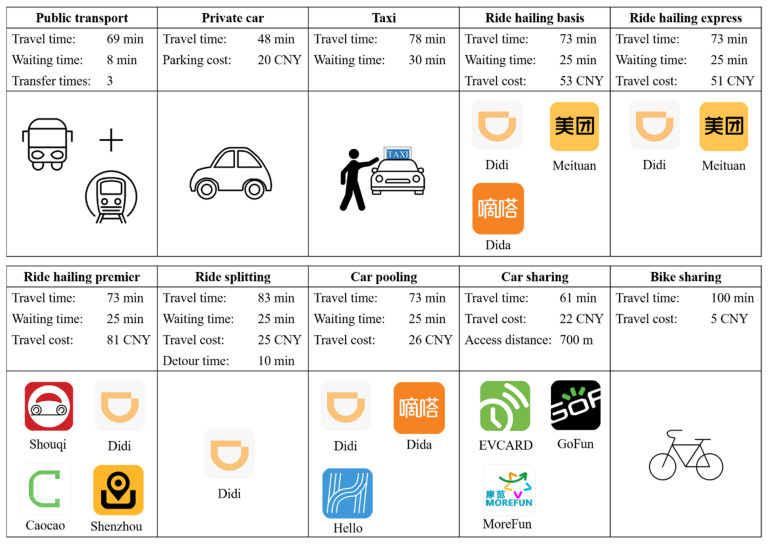
An example of the SP experiment (under the scenario of leisure trip, evening peak, and 20 km).

Based on these data, the SP experiment takes on an orthogonal design, an effective scenario reduction method compared to a full factorial design. With SPSS 21 software, 36 final scenarios are obtained. To enable respondents to distinguish between the shared modes, each scenario includes the travel costs. The travel time corresponding to distance is also added. In summary, a diagram of the SP experiment is shown in Figure 2. For each scenario, we, respectively, investigate travel mode choice before and during the COVID-19 pandemic. The pandemic began around the Spring Festival, a traditional Chinese festival on 24 January 2020. Thus, we define “before the COVID-19 pandemic” as “before 24 January 2020”. According to [55], all communities in Beijing had become low-risk areas as of 20 July 2020. Then, we define “during the COVID-19 pandemic” as “24 January–20 July 2020”. To lighten the answering burden, three SP scenarios were randomly assigned to every respondent.

#### 3.2.2. Participants

We carried out the survey in Beijing in December 2020 with the assistance of a professional online survey company in China. By this time, the population had experienced the emergency and normalization phases of the pandemic, thus people had formed specific travel habits during the COVID-19 pandemic. In order for the sample to be representative, the survey follows a spatially stratified random sampling strategy covering the 16 districts in Beijing. The respondents were spread across the city and each district has homogeneity with respect to the intensity of shared mobility use.

To calculate the minimum sample size before initiating the investigation, we used the suggestion by Orme [56], a commonly employed rule of thumb, specified as

(1) $$N\geq 500\cdot \frac{L}{J\cdot S}$$

where L is the largest number of levels for any attributes, J is the number of alternatives, and S is the number of choice scenarios faced by each respondent. Following this rule, the sample size of this survey would be larger than 84 ((500 × 5)/(10 × 3)). Additionally, Lancsar and Louviere [57] suggested 20 respondents for each choice scenario, thus the sample size required was 240 (20 × 36/3). 

Respondents who have used the aforementioned shared mobility services were selected from permanent inhabitants in Beijing. According to the following principles, 1007 valid questionnaires were collected: (1) respondents lived in Beijing in both the pre- and post-pandemic periods; (2) all questions were answered in full; (3) results from similar questions are consistent. This resulted in 3021 mode choice data, with approximately 83 or 84 observations obtained for each SP scenario. 

Table 2 summarizes the socio-demographic characteristics of participants. The gender distribution of the sample is consistent with that of the whole population in Beijing [58]. There are fewer elderly over 50 in our sample, because they are not proficient in the use of online surveys and shared mobility apps. This result is consistent with low participation among the elderly in terms of both the internet and shared mobility services [7,59,60]. More than 80% of the respondents do not have school-age children. Additionally, the car ownership rate in the sample is 9.36% higher than the average level in Beijing.

The perceived severity of the pandemic among different populations is also presented in Table 2. Men and women have a similar perception of the severity of the pandemic. The elderly take the pandemic more seriously than the young. Respondents with cars and school-age children pay more attention to the severity of the pandemic.

Additionally, we collected the number of accumulated COVID-19 confirmed cases in participants’ residential districts by 20 July 2020. The subjective perception and objective confirmed cases are considered together to characterize the severity of the pandemic more completely.

#### 3.2.3. Attitudes toward Shared Mobility Services

Shared mobility is associated with instrumental value, technology adoption, openness to innovation, lifestyle, and other awareness [3,13,61,62]. As such, different questions were used to investigate and quantitatively measure attitudes. Respondents were asked to express how much they agreed with statements about shared mobility use on five-point Likert scales, with 1 symbolizing “completely disagree” and 5 symbolizing “completely agree”. The description of these statements is listed in Table 3. Cronbach’s alpha of the statements is 0.752, higher than 0.7. Thus, the internal consistency and reliability of the scale are acceptable [63,64].

**Figure 3:**
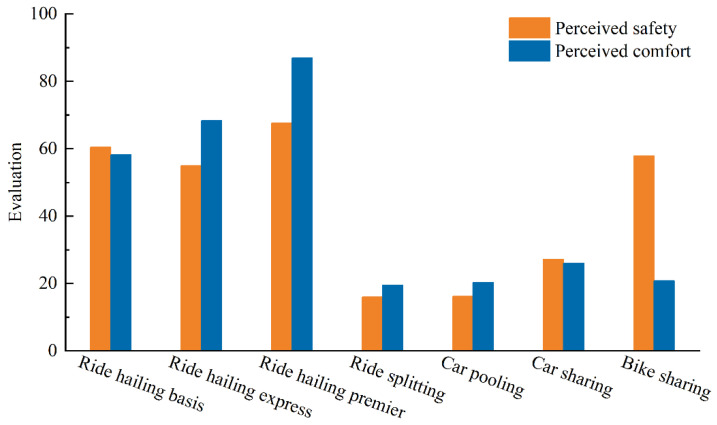
The perceived safety and comfort of different shared mobility services.

For each shared mobility service, respondents needed to evaluate its safety and comfort on a scale from 0 to 100. The average scores are clearly shown in Figure 3. The order of perceived safety is: ride hailing > bike sharing > car sharing > ride sharing; and the order of perceived comfort is: ride hailing > car sharing > bike sharing > ride sharing. In terms of ride hailing services, the safety of ride hailing express is lower than that of ride hailing basis, while the comfort of the former is higher. Ride hailing premier is judged to be the safest and most comfortable service, while ride sharing is the opposite. Both the safety and comfort of car sharing need to be improved. In particular, bike sharing is an alternative with high safety and low comfort.

### 3.3. Latent Class Analysis

LCA is a method used to discern any meaningful and scientifical latent classes against the noise error [65]. The latent variable is measured indirectly by two or more manifest variables. The indicators and the latent variable are both treated as categorical, which is a clear distinction between LCA and other latent variable models (e.g., factor analysis, latent trait analysis, and latent profile analysis).

Suppose that D numbers of observed variables are used to identify C classes of the latent variable X. Indicator d has $l_{d}=1,\;2,\ldots ,\;L_{d}$ categories. Each individual only belongs to one class c, thus the sum of each class’s prevalence is equal to 1. The response probabilities corresponding to each category of indicators also sum to 1, expressed as Equations (2) and (3).

(2) $$\sum_{c=1}^{C}P\left(X=c\right)=1$$

(3) $$\sum_{l_{d}=1}^{L_{d}}P(d=l_{d}|X=c)=1$$

Let $y=l_{1},l_{2},\ldots ,\;l_{D}$ refer to the vector of each response to D indicators. The combination of $\prod_{d=1}^{D}L_{d}$ numbers of y is the array of response patterns, represented by Y. Establish a dummy variable $\gamma_{d}$ equal to 1 when the response to observed variable d is $l_{d}$, otherwise 0. Based on the fundamental assumption of local independence, the conditional probability of a particular response y within class c is specified as

(4) $$P(Y=y|X=c)=\prod_{d=1}^{D}\prod_{l_{d}=1}^{L_{d}}P{\left(d=l_{d}|X=c\right)}^{\gamma_{d}}$$

The marginal probability of response y is formulated as

(5) $$P\left(Y=y\right)=\prod_{c=1}^{C}P\left(X=c\right)\prod_{d=1}^{D}\prod_{l_{d}=1}^{L_{d}}P{\left(d=l_{d}|X=c\right)}^{\gamma_{d}}$$

The classification probability conditional on response pattern y is obtained using Bayes’ theorem, stated as

(6) $$P(X=c|Y=y)=\frac{P(Y=y|X=c)P\left(X=c\right)}{P\left(Y=y\right)}$$

In this study, the latent variable is the shared mobility pattern. The observed indicators (each with five categories) are V1–V9 in Table 3. For each individual, the probability belonging to each class of latent variable is calculated by Equation (6) based on its own response.

### 3.4. Nested Logit Model

**Figure 4:**
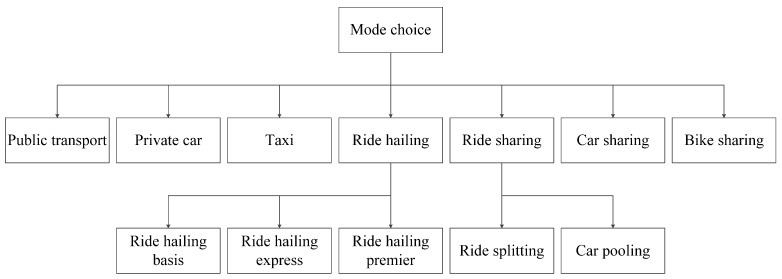
NL model structure.

Discrete choice models are statistical methods to examine the utility-maximizing choice behavior by the decision maker [66]. To capture the correlated unobserved effects among different shared mobility services, NL models are, respectively, applied before and during the COVID-19 pandemic. The choice set is partitioned into K non-overlapping nests. As shown in Figure 4, the two-level structure consists of 10 alternatives and 7 nests. Ride hailing basis, ride hailing express, and ride hailing premier are placed into the “ride hailing” nest, while ride splitting and car pooling are added to the “ride sharing” nest. Alternatives in different nests are independent of each other, and those in the same nest are correlated. IIA holds within each nest but not across nests.

The utility $U_{nj}$ that traveler n obtains from mode j in nest $B_{k}$ is specified as

(7) $$U_{nj}=V_{nj}+\varepsilon_{nj},\;j\in B_{k}$$

where $V_{nj}$ is the representative utility associated with observed attributes; $\varepsilon_{nj}$ captures the factors affecting the utility but is not included in $V_{nj}$. $V_{nj}$ can be decomposed as $W_{nk}$ (constant for all alternatives within nest $B_{k}$) and $Y_{ni}$ (varying over alternatives within a nest). The two parts coincide with the linear form, specified as

(8) $$W_{nk}=\beta_{k}X_{k}$$

(9) $$Y_{ni}=\beta_{i}X_{i}$$

where $X_{k}$ and $X_{i}$ are the explanatory variable vectors for mode i and nest $B_{k}$, respectively; $\beta_{k}$ and $\beta_{i}$ are the corresponding coefficient vectors. 

The marginal probability $P_{nB_{k}}$ that a mode in nest $B_{k}$ is chosen and the conditional probability $P_{ni|B_{k}}$ of choosing mode i within nest $B_{k}$ is expressed as Equations (10) and (11). Multiply $P_{nB_{k}}$ by $P_{ni|B_{k}}$, the probability $P_{ni}$ of traveler n chooses mode i is formulated as Equation (12).

(10) $$P_{nB_{k}}=\frac{exp\left(W_{nk}+\lambda_{k}I_{nk}\right)\;}{\sum_{l=1}^{K}exp\left(W_{nl}+\lambda_{l}I_{nl}\right)\;}$$

(11) $$P_{ni|B_{k}}=\frac{exp\left(Y_{ni}/\lambda_{k}\right)}{\sum_{j\in B_{k}}exp\left(Y_{nj}/\lambda_{k}\right)},\;i\in B_{k}\;$$

(12) $$P_{ni}=P_{nB_{k}}P_{ni|B_{k}}$$

Note that $I_{nk}=\ln\sum_{j\in B_{k}}exp\left(Y_{nj}/\lambda_{k}\right)$ is the inclusive value of nest $B_{k}$. $\lambda_{k}$ is the measurement of independence among the modes within nest $B_{k}$. As $\lambda_{k}$ increases to 1, the correlation will drop to zero.

For explanatory variables in NL models before and during the COVID-19 pandemic, the mode-specific variables and the safety and comfort of different shared mobility services are positioned at lower level. The socio-demographics, trip context variables, and the latent shared mobility use pattern derived from LCA are placed at the upper level as they vary with individuals and SP scenarios. The COVID-19–related characteristics (e.g., the perceived severity of the pandemic and confirmed cases) are specifically for the during-pandemic model.

## 4. Results

### 4.1. Travel Mode Choice Distribution

**Figure 5:**
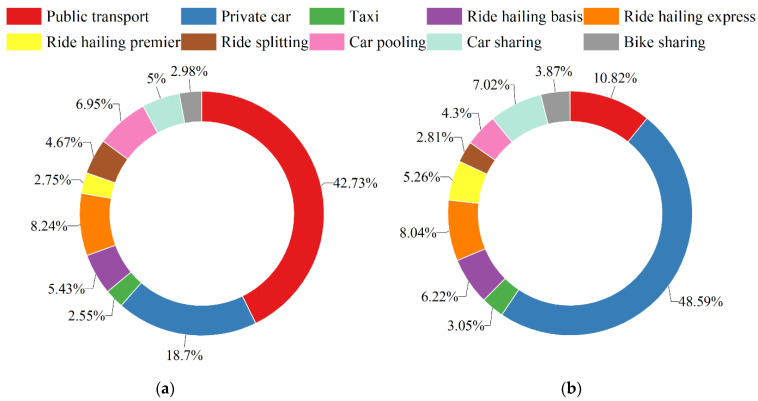
Travel mode choice before and during the COVID-19 pandemic. (**a**) Before the COVID-19 pandemic. (**b**) During the COVID-19 pandemic.

Travel mode choice among respondents is displayed in Figure 5. Before the COVID-19 pandemic, public transport was the primary mode, 42.73%, followed by private car, 18.70%. during the COVID-19 pandemic, private car is most favored, 48.59%, and public transport use drops sharply to 10.82%. The mode split of shared mobility is 36.01% and 37.54%, respectively, before and during the pandemic. 

The popularity of shared mobility services is sorted as: ride hailing express > car pooling > ride hailing basis > car sharing > ride splitting > bike sharing > ride hailing premier, and ride hailing express > car sharing > ride hailing basis > ride hailing premier > car pooling > bike sharing > ride splitting during the extraordinary times. The proportion of ride sharing decreases 4.50%, while that of ride hailing, car sharing, and bike sharing increases by 3.11%, 2.02%, and 0.89%. Hence, people prefer low-occupancy vehicles with stronger controllability of COVID-19 risk.

### 4.2. Latent Class Analysis Results

We first determine the optimal number of latent classes. A series of models with two to five classes are estimated, and the fit statistics are presented in Table 4. The two-class solution is selected because both Akaike’s information criterion (AIC) and the Bayesian information criterion (BIC) are the lowest, and the sample size in each cluster is relatively balanced.

**Figure 6:**
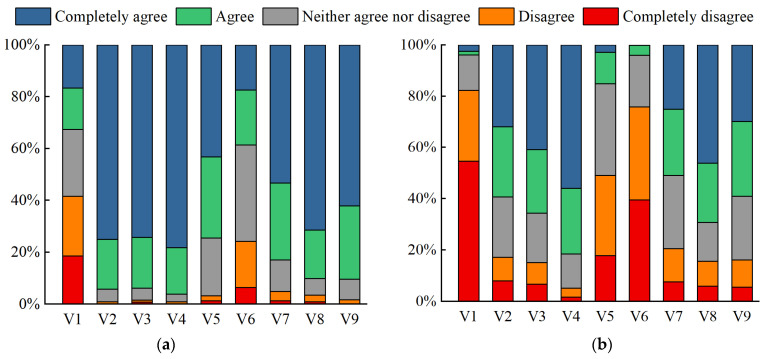
The conditional probability within two latent classes. (**a**) Class 1: shared-mobility optimists. (**b**) Class 2: shared-mobility pessimists.

The conditional probability of each observed variable is graphically shown in Figure 6. The probability related to “disagree” for class 2 is higher than that for class 1. Therefore, the individuals can be classified as shared-mobility optimists and shared-mobility pessimists, accounting for 57% and 43%, respectively.

### 4.3. Nested Logit Model Estimation Results

The package PandasBiogeme 3.2.6 was used to estimate the parameters in NL models. The multiple synteny problems are found to be non-existent. Several variables are insignificant for all alternatives, therefore are removed from the models. The estimation results are shown in Table 5. The goodness-of-fit measures, $\rho^{2}$, are 0.267 and 0.301. The scale parameters before and during the COVID-19 pandemic (0.714/0.699 and 0.613/0.606) are between 0 and 1, thus the nested structure is rational.

Different factors that influence shared mobility use before and during the COVID-19 pandemic:

(1) Waiting time: due to the panic about the exposure to public places during the pandemic, people are more sensitive to a longer waiting time, thus this variable becomes more influential.

(2) Transfer times: during the pandemic, transfer times have a more negative effect on the use of public transport.

(3) Parking cost: the use of private cars has risen due to COVID-19, thus the parking cost has a more negative effect.

(4) Detour time: the pre-pandemic ride splitting choice is negatively affected by the detour time. During the COVID-19 pandemic, the anxiety about shared spaces becomes more important, and the detour time is insignificant.

(5) Access distance: car sharing with lower close contact risk becomes more popular during the pandemic. Therefore, the negative effect of the access distance decreased.

(6) Travel cost, safety and comfort: travel cost is less influential in the COVID-19 context due to decreased travel demand. Perceived safety and comfort are more concerning when using shared mobility. 

(7) School-age children: families with school-age children have lowest preference for bike sharing and public transport, followed by ride sharing and ride hailing. Hence, these families are less likely to choose high-occupancy vehicles. Impacted by the disruptive pandemic, children study at home, and family outdoor activities have no correlation with children going to school. Then, the related coefficients are all insignificant. 

(8) Car ownership: the pandemic has strengthened the positive effect of this variable on private car use. Further, ride hailing and ride sharing are favored by car owners during the COVID-19 pandemic, thus these services can alleviate the intensity of private car use.

(9) Transportation terminal access: travelers leaving for the transportation terminal are less likely to use private cars. The preference for private cars is also lower than that for car sharing in such terminal-related trips during the COVID-19 pandemic.

(10) Shared-mobility optimists: the pandemic has changed the effect of this variable on ride sharing from positive to negative, and has decreased the negative effect on private cars. Thus, the shared-mobility pessimists would show a modal shift from public transport to ride sharing, and the car dependency of shared-mobility optimists would increase. 

(11) Subjective and objective severity of the pandemic: the severity of the pandemic is directly reflected in the number of confirmed cases, although this statistical indicator is not identical to the perceived severity of the pandemic. The latter is associated with prevention strategies in the region and elsewhere, and thus more complicated. Perceived severity is negatively correlated with the use of public transport and ride sharing, and is positively correlated with private car use, car sharing, and bike sharing. In comparison, the confirmed cases exhibit trivial effects on travel mode choice.

## 5. Discussion

### 5.1. Relationship between Perceived COVID-19 Severity and Travel Mode Choice

**Figure 7:**
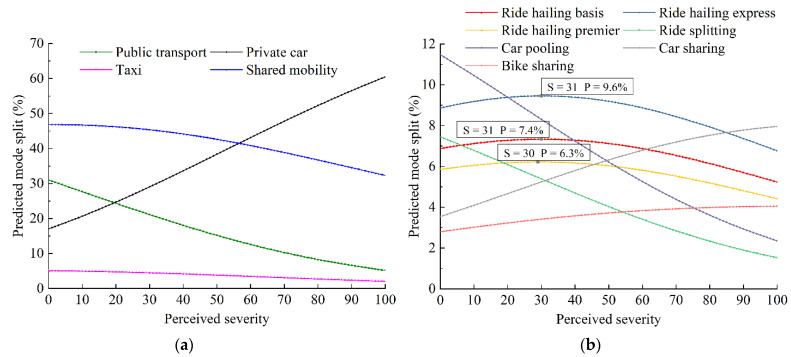
Predicted travel mode split in response to the perceived severity of the pandemic. (**a**) Conventional and shared mobility. (**b**) Shared mobility services.

The predicted travel mode split with the gradual perceived COVID-19 severity is illustrated in Figure 7. As shown in Figure 7a, the preference for public transport decreased from 31.00% to 5.18%, while that for private cars increased from 17.04% to 60.47%. The mode split of shared mobility decreased, and the decline rate will increase as the pandemic worsens. 

A detailed analysis for each shared mobility service is carried out, as shown in Figure 7b. When the perceived COVID-19 severity is less than 30, travelers exhibit a growing preference for ride hailing. The maximum choice probabilities of ride hailing basis, ride hailing express, and ride hailing premier are 7.4%, 9.6%, and 6.3%, respectively. In contrast, the ride sharing split shows a continuous reduction. Additionally, preference for car sharing and bike sharing increased, thus these two mobility services have great potential in the COVID-19 context.

### 5.2. Relationship between COVID-19 Severity and Price Discount for Shared Mobility

As shown in Figure 7b, ride hailing and ride sharing are vulnerable to the pandemic. To alleviate the negative effects of COVID-19 on the shared mobility ridership, pricing management strategies need to be implemented by transportation network companies. We predict the mode split of specific mobility services in response to differences in the perceived severity of the pandemic and price discounts, and then explore the price discount needed to maintain the ridership despite the impact of COVID-19. 

**Figure 8:**
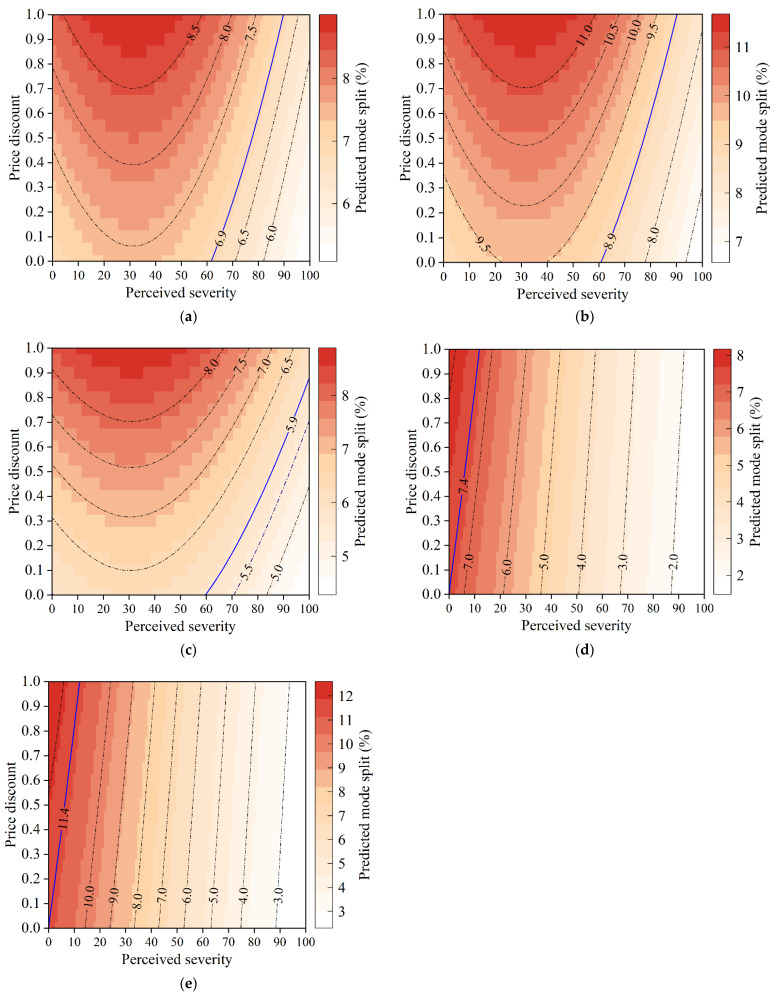
Mode split changes in ride hailing and ride sharing. (**a**) Ride hailing basis. (**b**) Ride hailing express. (**c**) Ride hailing premier. (**d**) Ride splitting. (**e**) Car pooling.

Figure 8 demonstrates the mode split changes in ride hailing and ride sharing and mode split values on the same contour line are equal. For ride hailing, the mode split of ride hailing basis, ride hailing express, and ride hailing premier is 6.9%, 8.9%, and 5.9%, respectively, when the perceived severity of the pandemic is 0. As displayed in Figure 8a–c, when perceived severity exceeds 60, a price discount is required since the mode split begins to decrease. A discount of 10% off is recommended at a perceived severity of 65. When the perceived severity increases to 80, discounts of 60%, 60%, and 40% off are recommended for the three services, respectively. When the pandemic is at its worst, only ride hailing premier can maintain its ridership with 90% off. 

For ride sharing, the mode split of ride splitting and car pooling is 7.4% and 11.4%, respectively, when the perceived severity of the pandemic is 0. A price discount plays a limited role in maintaining such a ridership, and will be totally ineffective with a perceived severity over 12. This may be because: (1) the price discount has little effect on the adoption of lower-cost services; and (2) it is more difficult to maintain social distancing in high-occupancy vehicles and they are thus less acceptable in the COVID-19 context.

## 6. Conclusions

This study provides insights into the impact of COVID-19 on travel mode choice in terms of shared mobility. Four types of shared mobility services were considered, including ride hailing, ride sharing, car sharing, and bike sharing. Based on travel mode choice data collected from a stated preference survey, we found that public transport was the primary mode before the COVID-19 pandemic, and its mode split dropped sharply from 42.73% to 10.82% due to the pandemic. Private car is most favored during the COVID-19 pandemic, and its mode split has increased from 18.70% to 48.59%. The mode split of shared mobility is always between 36% and 38%. Among mobility services, the proportion of ride sharing choice decreased by 4.50%, while that of ride hailing, car sharing, and bike sharing increased by 3.11%, 2.02%, and 0.89%.

The latent class analysis and nested logit models were combined to compare travel mode choice before and during the COVID-19 pandemic. The estimates show that with the transition from public to private transport, shared mobility has the potential to avoid the high transmission risk of public transport and also alleviate the intensity of private car use during the COVID-19 pandemic, but this is limited by anxiety about shared spaces. Moreover, the subjective perceived severity of the pandemic has stronger effects on travel mode choice behavior than objective confirmed cases. As perceived severity increases, preference for ride hailing and ride sharing decreases, so a price discount for ride hailing is effective at maintaining the ridership despite the impact of COVID-19. 

These findings are beneficial in developing appropriate strategies for shared mobility services to adapt to the pandemic—for example, by developing complete in-vehicle prevention procedures, including sanitizing regularly, providing masks and disinfectant, and recording passengers’ health conditions. For each perceived severity level, shared mobility services can make adaptive restrictions on the ridership and share duration of ride sharing through their platforms. Severity-driven pricing management strategies are needed to develop appropriate strategies for shared mobility services to adapt to the pandemic.

There are still limitations to this study that are worth highlighting in further research. Using the empirical evidence based on SP data may lead to discrepancies between respondents’ answers and their actual behavior. Further research will make attempts to combine the survey data and big data to improve the reliability of travel mode choice analysis. Additionally, we would like to take post-COVID-19 travel mode choice behavior into consideration, developing appropriate strategies for shared mobility services to adapt to the pandemic.

## Figures and Tables

**Table 1:** Summary of factors that influence shared mobility use in the literature.

Factor	Ride Hailing	Ride Sharing	Car Sharing	Bike Sharing	Source
Male	-	-	+	+	[3,12,13]
Age	-	+	-	+	[17,18,19,20]
Income	+	-	+	+	[21,22,23]
Education	+	-	+	+	[2,20,22,23]
Car ownership	-	-	-	+	[7,22,23,24]
Security risk	-	-	-	-	[25,26,27,28]
Environmental awareness	+	+	+	+	[3,14,29,30]
Bad weather	+	+	+	-	[15,31,32,33]
Non-commuting	-	+	+	-	[2,5,7,8]
Travel time	-	-	-	-	[2,7,20,31]
Travel cost	-	-	-	-	[2,7,16,31]

Note: “+” indicates a positive relationship, and “-” indicates a negative relationship.

**Table 2:** Statistics of participants and their perceived severity of the pandemic.

Variable	Description	Percentage (%)	The Whole Population of Beijing (%)	Perceived COVID-19 Severity
Gender	Male	52.63	51.63	72.2
	Female	47.37	48.37	72.04
Age (years)	18–29	37.64	20.21	68.09
	30–39	36.54	23.9	73.14
	40–49	19.36	18.49	76.25
	≥50	6.46	37.4	79.02
School-age children	Have 7-to-11-year-old children	14.9	–	71.39
	No 7-to-11-year-old child	85.1	–	76.31
Car ownership	One or more cars	63.36	54	62.45
	No car	36.64	46	77.71

**Table 3:** Statistics of attitudinal statements.

NO.	Statements	Mean	SD
V1	I would like to choose departure time and travel routes flexibly.	4.526	0.801
V2	I am familiar with using smartphone apps to manage my trip.	4.265	1.082
V3	Shared mobility could alleviate the traffic congestion.	4.264	1.067
V4	I prefer to use shared mobility in the cases of parking difficulty and high parking fees.	4.186	1.095
V5	I can easily afford travel costs of shared mobility.	4.102	1.049
V6	I would like to take initiative to discover and try something new.	3.903	1.142
V7	I think shared mobility travel is more convenient than driving.	3.345	1.259
V8	I think shared mobility travel is more comfortable than driving.	2.592	1.221
V9	I think shared mobility travel is safer than driving.	2.311	1.304

**Table 4:** LCA fit statistics.

Number of Classes	Log Likelihood	AIC	BIC	*p*-Value	Group Size
2	−12,588.489	25,292.979	25,578.033	0.000	578/429
3	−12,767.084	25,630.168	25,866.076	0.000	158/548/301
4	−12,936.093	25,948.186	26,134.946	0.000	546/105/80/276
5	−13,158.101	26,372.203	26,509.815	0.000	24/86/169/254/474

**Table 5:** Estimated results of NL models.

Variable	Before COVID-19		During COVID-19		Variable	Before COVID-19		During COVID-19	
	Estimates	*p*-Value	Estimates	*p*-Value		Estimates	*p*-Value	Estimates	*p*-Value
Lower level					Constant				
Mode-specific variables					Public transport	2.860	***	2.080	***
Waiting time	−0.013	***	−0.025	***	Private car	1.620	***	0.837	**
Transfer times	−0.084	***	−0.102	*	Taxi (reference)	0		0	
Parking cost	−0.014	*	−0.019	***	Ride hailing basis	0.401		−0.489	
Detour time	−0.016	*	−0.011		Ride hailing express	0.863	**	−0.220	
Access distance	−0.480	***	−0.452	***	Ride hailing premier	−0.178		−0.643	
Travel cost	−0.006	***	−0.004	***	Ride splitting	0.727		0.256	
Perceived safety	0.008	***	0.009	***	Car pooling	0.817	**	0.432	
Perceived comfort	0.001		0.003	**	Car sharing	0.478		−0.909	*
					Bike sharing	3.130	***	2.070	***
Upper level									
School-age children (base: No 7-to-11-year-old child)					Shared-mobility optimists (base: shared-mobility pessimists)				
Public transport	−0.906	***	−0.206		Public transport	0.068		−0.271	
Private car	−0.417		−0.141		Private car	−0.508	**	−0.489	**
Taxi (reference)	0		0		Taxi (reference)	0		0	
Ride hailing	−0.565	*	0.052		Ride hailing	0.186		−0.106	
Ride sharing	−0.729	**	−0.069		Ride sharing	0.402	*	−0.526	**
Car sharing	−0.420		0.083		Car sharing	0.244		0.333	
Bike sharing	−1.070	**	−0.183		Bike sharing	−0.165		−0.314	
Car ownership (base: no car)					Perceived COVID-19 severity				
Public transport	−0.130		0.218		Public transport	–	–	−0.008	*
Private car	0.730	***	1.010	***	Private car	–	–	0.023	***
Taxi (reference)	0		0		Taxi (reference)	–	–	0	
Ride hailing	−0.050		0.416	*	Ride hailing	–	–	0.007	
Ride sharing	0.045		0.748	***	Ride sharing	–	–	−0.005	*
Car sharing	0.131		0.396		Car sharing	–	–	0.019	***
Bike sharing	−0.799	**	−0.061		Bike sharing	–	–	0.015	**
Transportation terminal access (base: not this trip purpose)					Confirmed cases				
Public transport	−0.504	**	−0.642	**	Public transport	–	–	0.001	
Private car	−0.621	**	−0.866	***	Private car	–	–	0.003	*
Taxi (reference)	0		0		Taxi (reference)	–	–	0	
Ride hailing	−0.053		−0.241		Ride hailing	–	–	0.002	
Ride sharing	−0.295		−0.279		Ride sharing	–	–	0.002	
Car sharing	−0.388		−0.532	**	Car sharing	–	–	0.003	
Bike sharing	−0.389		−1.250	***	Bike sharing	–	–	0.002	
Scale parameter									
$\lambda_{\mathrm{RH}}$	0.714	***	0.613	***	Log likelihood	−5018.882		−4736.341	
$\lambda_{\mathrm{RS}}$	0.699	**	0.606	**	$\rho^{2}$	0.267		0.301	
Sample size	3021		3021						

Note: Variable is not available in the model. *** Statistically significant at 0.01. ** Statistically significant at 0.05. * Statistically significant at 0.1.

## Data Availability

Due to privacy issues, the data may not be shared publicly.
